# Efficacy and Safety of Isoprenaline during Unstable Third-Degree Atrioventricular Block

**DOI:** 10.3390/jcdd10120475

**Published:** 2023-11-25

**Authors:** Manuel De Lazzari, Nicolò Martini, Federico Migliore, Filippo Donato, Luciano Babuin, Giuseppe Tarantini, Martina Perazzolo Marra, Luisa Cacciavillani, Emanuele Bertaglia, Andrea Bortoluzzi, Vito Cianci, Domenico Corrado, Sabino Iliceto, Alessandro Zorzi

**Affiliations:** 1 Department of Cardio-Thoraco-Vascular Sciences and Public Health, University Hospital of Padua, Via Giustiniani, 2, 35128 Padua, Italy; manuel.delazzari@aopd.veneto.it (M.D.L.); nicolo.martini.2@studenti.unipd.it (N.M.); federico.migliore@unipd.it (F.M.); filippo.donato@aopd.veneto.it (F.D.); luciano.babuin@aopd.veneto.it (L.B.); giuseppe.tarantini.1@unipd.it (G.T.); martina.perazzolomarra@unipd.it (M.P.M.); luisa.cacciavillani@aopd.veneto.it (L.C.); emanuele.bertaglia@aopd.veneto.it (E.B.); domenico.corrado@unipd.it (D.C.); sabino.iliceto@unipd.it (S.I.); 2 Emergency Department, Department of Medicine, University Hospital of Padua, Via Giustiniani, 2, 35128 Padua, Italy; andrea.bortoluzzi@aopd.veneto.it (A.B.); vito.cianci@aopd.veneto.it (V.C.)

**Keywords:** complete atrioventricular block, isoprenaline, dopamine, intensive care unit

## Abstract

Unstable and symptomatic complete atrioventricular block represents a potentially fatal condition that requires prompt therapy while waiting for definitive pacemaker implantation. Although transcutaneous pacing is included in acute management, it could be a difficult approach due to its painfulness and the occasional failure of mechanical capture. Drug therapy is a feasible choice, and current guidelines encompass the use of atropine, dopamine, or epinephrine. Isoprenaline has never been investigated in this setting, and no specific indication of its use has been provided despite its potentially more favorable pharmacological profile. The study population included a consecutive series of patients who presented to the emergency department because of unstable third-degree atrioventricular block and were treated with either isoprenaline or dopamine infusion while waiting for definitive pacemaker implantation. Asymptomatic patients or those with reversible causes of complete atrioventricular block were excluded. The clinical response to the drug was deemed poor if, despite achieving a full drug dose, patients remained symptomatic and/or with hemodynamic instability, ventricular rate and rhythm did not improve or worsened, including if ventricular arrhythmias or asystolic pauses and/or irrepressible nausea/vomiting occurred. Isoprenaline infusion has proved to be safe and tolerated with no arrhythmia induction or hypotensive issues. Isoprenaline has also proven to be more satisfactory in achieving an effective clinical response in 84% of patients rather than dopamine (31%, *p* < 0.001), reducing the need for temporary artificial pacing. Our data point out the efficacy and safety of isoprenaline infusion and its greater tolerability over dopamine in the acute management of unstable third-degree AV block while waiting for definitive pacemaker implantation.

## 1. Introduction

Third-degree atrioventricular (AV) block is a complete loss of communication between the atrium and ventricle through the regular cardiac electroconductive pathways. Without appropriate conduction through the AV node, the sinoatrial node cannot act to control the heart rate, leading to a reduction in cardiac output up to an asystolic cardiac arrest. The decreasing cardiac output may lead to hemodynamic instability and symptoms such as hypotension, syncope, altered consciousness, angina, and heart failure. The condition can be fatal if not promptly treated. Current American guidelines recommend a specific treatment only for symptomatic and unstable bradycardic rhythms [1,2]. First-line therapy includes the intravenous administration of Atropine, even if it is unlikely to improve the AV block at the His bundle or His-Purkinje level, and isolated reports have suggested occasionally worsened AV conduction and/or hemodynamic compromise in such patients. However, because of its short duration of action, it is generally used as a bridge to longer-lasting therapy, such as beta-adrenergic agonists (class of recommendation II evidence B) or transcutaneous and eventually transvenous pacing (IIa) [1]. Recently, the Advanced Cardiac Life Support (ACLS) Guidelines provided by the American Heart Association recommend, if atropine is not effective, to consider transcutaneous pacing and/or dopamine IV infusion (5 to 20 mcg/kg per minute) or epinephrine (2–10 mcg per minute) [2]. The efficacy of dopamine was shown to be equal to transcutaneous pacing (TCP) in patients with unstable bradycardia unresponsive to atropine in a small size sample in the prehospital setting [3]. Despite its effectiveness, three major adverse events were recorded, including ventricular tachycardia, ventricular fibrillation, and cardiac arrest. Epinephrine and dobutamine are mainly administered in specific circumstances other than bradycardia (cardiac arrest, heart failure, or cardiogenic shock). Isoprenaline is a non-selective beta-adrenoreceptor agonist that was shown to elicit an escape rhythm in pacemaker-dependent patients undergoing generator replacement [4]. Despite its chronotropic positive effect, no specific indication of its use in this setting of patients has been provided in current ACLS guidelines, probably due to its mild hypotensive effect caused by the lowering of peripheral vascular resistances.

Our study aimed to assess the safety and the efficacy of isoprenaline infusion as an alternative to dopamine in a consecutive series of patients admitted to the critical care unit for unstable complete AV block while waiting for definitive pacemaker implantation.

## 2. Methods Section

### 2.1. Study Design

This study is a retrospective, descriptive study reviewing consecutive patients presented to the emergency department (ED) from January 2017 to November 2021 who were diagnosed with an unstable third-degree AV block. According to guidelines, an unstable third-degree AV block is defined in the presence of systolic blood pressure less than 90 mmHg, ischemic chest pain, dyspnea, syncope, pre-syncope or altered mental status [1,2,5]. Patients with reversible causes of complete AV block (i.e., electrolyte disturbances, drug overdose, hypoxia, and cardiac ischemia) were excluded from the study after a comprehensive clinical workup that included clinical, anamnestic, laboratory, electrocardiographic, and pharmacological data [6]. Similarly, patients with asymptomatic third-degree AV block (not requiring therapy) were ruled out. According to local protocols, each patient had been provided with pacing pads of the external defibrillator and received urgent medical treatment to minimize/avoid artificial pacing according to the physician’s preference (i.e., isoprenaline or dopamine infusion).

### 2.2. Clinical Endpoints

The clinical response to treatment was considered effective if the patient became asymptomatic after drug infusion and remained so until definitive pacemaker implantation. The clinical response was deemed poor if, despite achieving a full dose of the drug, patients remained symptomatic, and/or with hemodynamic instability [5], ventricular rate and rhythm did not improve or worsened, ventricular arrhythmias (VA) or asystolic pauses and/or irrepressible nausea/vomiting occurred.

### 2.3. Data Collection

The emergency department and hospital medical records, including all ECG tracing, were reviewed. To assure consistency, all data were collected independently on a preprinted data-collection form by two observers (N.M and F.D.), and ambiguous cases were reviewed by a third author (F.M.).

Age, gender, clinical and electrocardiographic features, pharmacological data, and hemodynamic parameters (i.e., mean arterial pressure and urinary output) were collected. The study population was divided into groups based on the chronotropic drug in use. 

The study was approved by the local institutional review board, and because of its retrospective nature, no consent was required. 

### 2.4. Statistical Analysis

Continuous variables are expressed as the median (1st and 3rd percentiles). Categorical variables are expressed as a percentage. Categorical variables were compared using the Chi-squared test or Fisher exact test when appropriate. A two-tailed probability value of 0.05 was considered statistically significant. All analyses were performed using SPSS software (version 26, SPSS Inc., Chicago, IL, USA).

## 3. Results

The demographic and clinical characteristics of the population are shown in Table 1. The study population consisted of 78 patients (39 female, 50%). Median age at presentation was 80 ± 10 years. Patients with complete AV block and atrial fibrillation were 17 (22%). Asystolic cardiac arrest was the first symptom in 12 subjects (15%), while complete AV block complicated by ventricular arrhythmias (i.e., frequent premature ventricular beats and non-sustained ventricular tachycardia) occurred in 4 (5%). Six patients (7%) required transcutaneous pacing meanwhile drug infusion started and subsequently stopped when the drugs took effect. Dopamine was the starting drug in 16 patients (21%) with a median dosage of 8 (IQ-iles 7–11) mcg/kg/min, while isoprenaline was used in 62 patients (79%) with a median dosage of 0.02 (IQ-iles 0.02–0.04) mcg/kg/min. Drug crossover/artificial pacing became necessary in 21 patients (27%) because of a poor clinical response (Table 2).

### 3.1. Clinical Response to Dopamine as First Drug

The clinical response was deemed effective by physicians only in 31% of patients, while the remaining showed a poor response (Table 2). The mean arterial pressure during drug infusion was 76 mmHg and remained substantially unchanged during the whole treatment. No patients required additional vasopressive agents. Clinical response occurred after a mean time of 30 ± 15 min. The heart rate increased on average from 34 to 52 bpm. One-to-one conduction during sinus rhythm or the normalization of ventricular rate during atrial fibrillation occurred in four subjects (25%). Urinary output was preserved in 15 patients (94%). No ventricular arrhythmias occurred during dopamine administration. Four patients (25%) had nausea and vomiting that were incoercible and required a switch in drugs with the complete resolution of symptoms. Eleven patients (69%) showed a poor clinical and chronotropic response with the persistence of symptomatic severe bradycardia and underwent successful drug switch/temporary artificial pacing. Specifically, of these eleven patients, nine of them (59%) underwent a drug switch, and all of them obtained an improvement in their heart rate up to 20 ± 5 bpm and progressive regression, or in some cases abolition, of bradycardic or drug-induced symptoms (nausea, altered mental status and dyspnea).

### 3.2. Clinical Response to Isoprenaline as First Drug

An effective clinical response was obtained in 52 (84%) patients, whereas 10 patients (16%) showed an unremarkable effect with significant symptomatic bradycardic rhythm and underwent temporary artificial pacing. Clinical response occurred after a mean time of 20 ± 10 min. The mean arterial pressure during drug infusion was 81 mmHg and remained substantially unchanged during the whole treatment. No patients required additional vasopressive agents. The heart rate increased on average from 34 to 67 bpm. Sinus rhythm with 1:1 AV conduction or atrial fibrillation with a normal ventricular rate occurred in 39 subjects (63%). Urinary output was preserved in 57 patients (92%) during isoprenaline infusion. No ventricular arrhythmias occurred. Of note, four patients presenting with an AV complete block and ventricular arrhythmias achieved a normalization of their heart rate with VA suppression after drug administration.

### 3.3. Comparison between Dopamine and Isoprenaline

**Figure 1:**
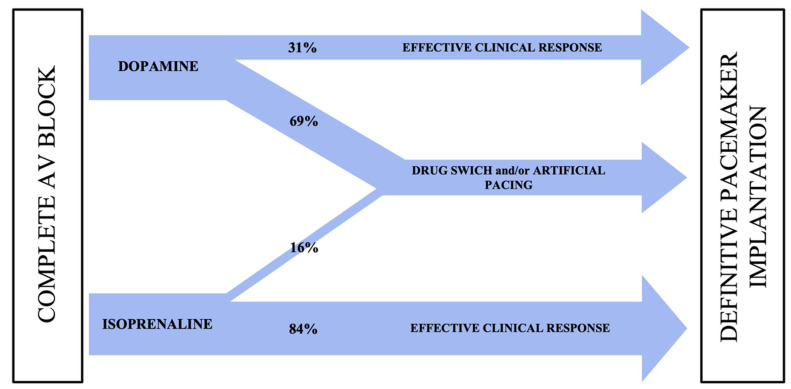
Dopamine and isoprenaline workflow. An effective clinical response occurred when the patient became asymptomatic and/or maintained hemodynamic stability with a preserved urinary output. Isoprenaline was statistically related to a more effective response than dopamine. Drug switch and/or artificial pacing were necessary more often in the dopamine group. AV: atrioventricular.

**Figure 2:**
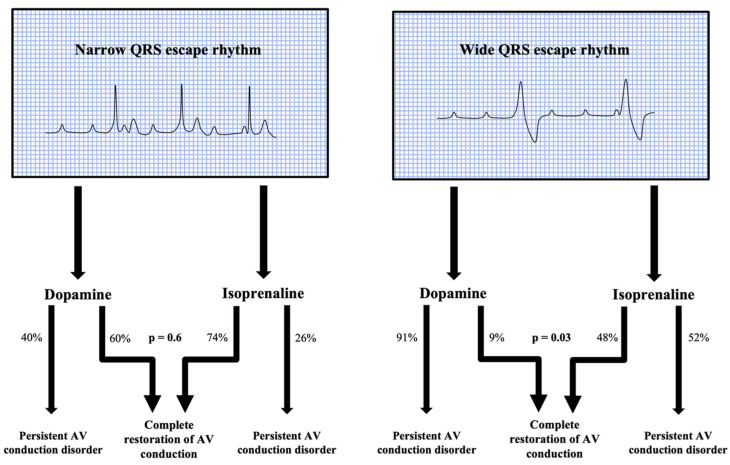
Effects of Isoprenaline and dopamine on AV conduction. The complete recovery of AV conduction occurred more frequently in narrow QRS patients. No differences were found between isoprenaline and dopamine in restoring normal AV conduction in this subset of subjects (*p* = 0.6). In patients presenting with a wide QRS escape rhythm, isoprenaline infusion was associated with complete recovery of normal AV conduction if compared to dopamine (*p* = 0.03).

**Figure 3:**
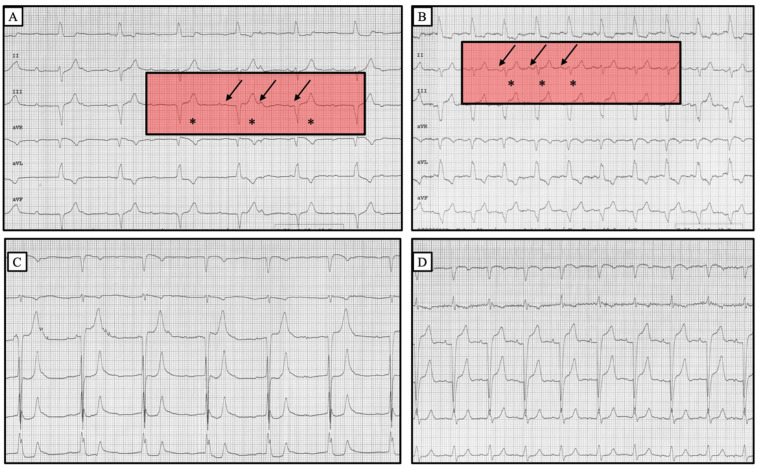
Twelve lead ECGs of a patient affected by an unstable complete AV block before (**A**,**C**) and after (**B**,**D**) isoprenaline infusion. A complete AV dissociation is appreciable in A. After isoprenaline administration, the patient regained 1:1 conduction (**C**). No significant variation in QRS complex morphology occurred before and after drug infusion. (Arrows indicate p waves and asterisks indicate QRS complexes).

**Figure 4:**
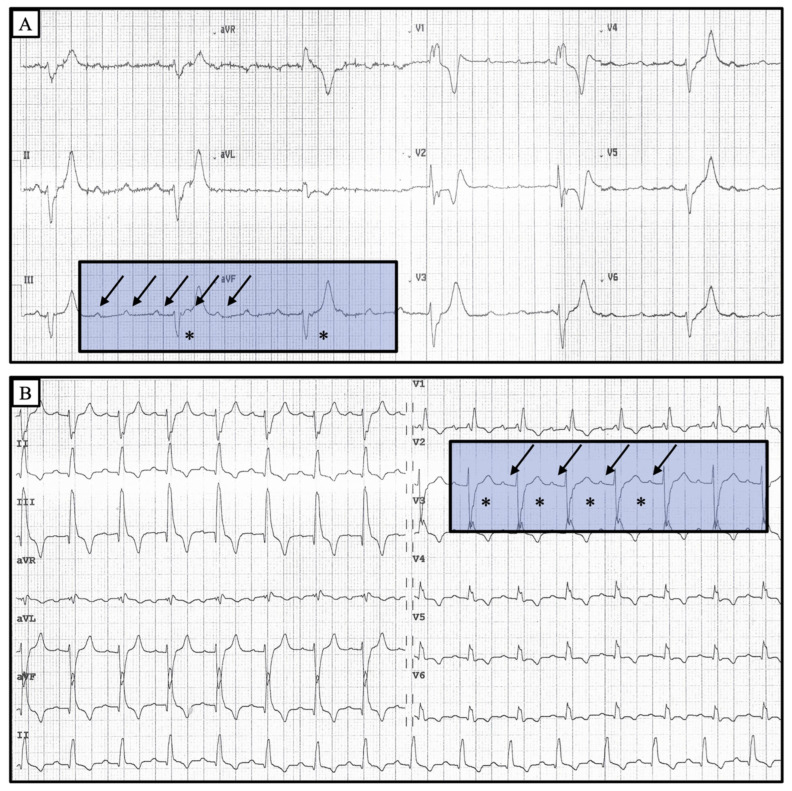
Twelve lead ECGs of a patient affected by an unstable complete AV block before (**A**) and after (**B**) isoprenaline infusion. A complete AV dissociation with the wide QRS escape rhythm is appreciable in A. After isoprenaline administration, the patient regained 1:1 conduction (**B**). Even though basal ECG was consistent with a trifascicular block (first degree AV block, right bundle branch block and posterior fascicular block), ECG at presentation clearly showed a ventricular escape rhythm given the marked slow rate, the wider QRS complexes and the different QRS axis. Arrows indicate p waves and asterisks indicate QRS complexes.

Isoprenaline infusion has proved to be effective and well tolerated (Figure 1, Table 2 and Table 3). After isoprenaline infusion, a satisfactory clinical response was obtained in 84% of patients versus 31% of subjects in the dopamine group (*p* < 0.001). Moreover, 39 patients (63%) in the isoprenaline-treated group showed the restoration of sinus rhythm with a 1:1 AV conduction or normal conducted atrial fibrillation compared to 4 (25%) in the dopamine group (*p* = 0.007). The complete recovery of AV conduction occurred more frequently in patients presenting with narrow escape QRS morphology rather than wide QRS morphology (*p* = 0.002). In the subset of patients with narrow QRS escape rhythm, no differences were found between isoprenaline and dopamine in restoring normal AV conduction. Of note, in the subset of patients presenting with a wide QRS escape rhythm, a complete recovery of normal AV conduction occurred more frequently in the isoprenaline group (48% patients vs. 9%, *p* = 0.03) (Figure 2, Figure 3 and Figure 4). 

No patients presented VA during beta-adrenergic infusion, not even with isoprenaline. Finally, none of the isoprenaline group presented incoercible nausea/vomiting during drug infusion compared to 25% of patients treated with dopamine (*p* = 0.02). 

## 4. Discussion

A complete AV block is considered a potentially life-threatening arrhythmia, with an overall prevalence of 0.04% worldwide [6], which, if not reversible, results from various pathologic states causing infiltration, fibrosis, or the loss of connection in portions of the healthy conduction system requiring permanent pacemaker implantation. In the case of a symptomatic, unstable AV block with hemodynamic compromise, prompt treatment becomes necessary while waiting for definitive pacing, and drug therapy is the most feasible choice. Current guidelines [2] encompass atropine and dopamine/epinephrine as the first line of therapy. Other beta-adrenergic agonists with a potentially more favorable pharmacological profile (i.e., isoprenaline) have never been investigated.

The main findings of this study are as follows: isoprenaline has proved to be effective in the acute management of symptomatic complete AV block despite not being planned in the current guidelines; isoprenaline presented a lower incidence of adverse effects than dopamine, and it reduced the need for temporary artificial pacing while waiting for definitive pacemaker implantation.

Dopamine is a well-recognized chronotropic drug, and it is considered the main therapy to treat unstable bradycardic rhythms as recommended by current guidelines [2]. This is mainly derived from a small randomized trial (82 patients) reporting that dopamine is equivalent to TCP in the emergency setting [3]. The only adverse effects analyzed were the occurrence of ventricular arrhythmias, without differences between the two groups, and the onset of chest burn/discomfort in a nonnegligible percentage of TCP patients. Given the dopamine mechanism of action, it is well known that this drug can be poorly tolerated and is responsible for the induction of nausea and vomiting, which is an eventuality that could further complicate resuscitation maneuvers or invasive procedures (i.e., definitive pacemaker implantation) [7]. Despite this, dopamine potentially has a more favorable vasopressive profile, as total peripheral resistance usually remains unchanged with low–intermediate doses of dopamine and decreases with higher doses [8]. Isoprenaline was shown to improve AV and intraventricular conduction in a small study on 37 patients with different degrees of AV block [9]. Moreover, it elicited the escape rhythms of pacemaker-dependent patients undergoing pacemaker replacement, minimizing the need for temporary transvenous pacemaker positioning [4]. The reported side effects of isoprenaline include ventricular arrhythmias, the worsening of myocardial ischemia, and hypotension due to a transient vasodilatation [1,10]. Our patients did not show any hypotensive episodes, and mean arterial pressures remained stable during drug infusion, similar to dopamine recipients. This mechanism is probably due to the fact that to have a positive chronotropic effect, sufficient low doses of isoprenaline, less than those required to induce clinically significant action on peripheral vascular resistance, are needed [11,12]. Instead, a chronotropic effect was elicited by dopamine after only an intermediate drug dosage, while the action in peripheral vascular resistance already began with lower doses due to dopaminergic activity. 

Our study results point out the efficacy of isoprenaline and its potential (over dopamine) for improving AV conduction, leading to hemodynamic stability and preserving urinary output without nausea/vomiting or any serious life-threatening side effects. Most of the patients treated with isoprenaline showed a good clinical outcome, and more than half regained normal AV conduction (Figure 2, Figure 3 and Figure 4). Furthermore, the benefit of isoprenaline over dopamine was more evident in those patients with a wide QRS escape rhythm. 

Additionally, patients presenting with AV block and VA that were treated with isoprenaline achieved the disappearance of the arrhythmic ventricular bradycardia-induced burden.

### Study Limitations

Given the retrospective nature of this observational monocentric study, it is not possible to evaluate the superiority, equivalency, or non-inferiority of isoprenaline. Of note, none of the other chronotropic drugs have been investigated in randomized controlled studies. This study may provide a realistic picture of the daily clinical practice in terms of the efficacy and safety of the acute management of unstable third-degree AV block, providing useful data for clinical practice. The sample size was not pre-determined, and patients were enrolled during a 5-year period consecutively. To minimize bias, their records were reviewed by experienced physicians independently using strict definitions in a preprinted data collection as stated in the Methods section. 

## 5. Conclusions

Although isoprenaline is not referred to in current guidelines, our data point out the efficacy and safety of isoprenaline infusion and its tolerability over dopamine in the acute management of unstable third-degree AV block while waiting for definitive pacemaker implantation.

## Figures and Tables

**Table 1:** Demographic and clinical characteristic of the study population (Total n = 78).

Characteristics	Overall	Dopamine Group	Isoprenaline Group	*p* Value
Male, n (%)	39 (50)	7 (44)	32 (52)	n.s.
Age (years)	81 (76–88)	86 (82–89)	79 (76–82)	0.012
Cardiac arrest at presentation, n (%)	12 (15)	3 (19)	10 (16)	n.s.
Features of unstable/symptomatic complete AV block				
- Systolic pressure <90 mmHg	27 (35)	6 (38)	21 (34)	n.s.
- Syncope/presyncope	22 (28)	6 (38)	16 (26)	n.s.
- Dyspnea	31 (40)	5 (31)	26 (42)	n.s.
- Chest pain/discomfort	6 (8)	2 (13)	4 (6)	n.s.
- Altered mental status	21 (27)	5 (31)	16 (26)	n.s.
Atrial fibrillation, n (%)	16 (21)	5 (31)	11 (18)	n.s.
Ventricular arrhythmias at presentation	4 (18)	0 (0)	3 (5)	n.s.
Median drug dosage (mcg/kg/min)		8 (7–11)	0.02 (0.02–0.04)	

Values are expressed as the number/total (percentage) of subjects or median (25th–75th percentiles).

**Table 2:** Clinical response to dopamine and isoprenaline.

Characteristics	Dopamine Group	Isoprenaline Group	*p* Value
Good clinical response, n (%)	5 (31)	52 (84)	<0.001
Poor clinical response, n (%)			
- Symptoms (dyspnea, chest pain, altered mental status)	8 (50)	8 (13)	n.s.
- Systolic pressure <90 mmHg	2 (13)	4 (6)	n.s.
- Syncope/presyncope	2 (13)	4 (6)	n.s.
- Lack of heart rate improvement/worsening heart rate	8 (50)	10 (16)	n.s.
Mean arterial pressure (mmHg)	76 (68–82)	81 (71–88)	n.s.
Preserved urinary output, n (%)	15 (94)	57 (92)	n.s.
Occurrence of ventricular arrhythmias, n (%)	0 (0)	0 (0)	n.s.
Nausea and vomiting, n (%)	4 (25)	0 (0)	0.02

Values are expressed as the number/total (percentage) of subjects or median (25th–75th percentiles). AV indicates atrioventricular.

**Table 3:** Hemodynamic parameters during effective response to the first drug.

Characteristics	Dopamine Group	Isoprenaline Group	*p* Value
Restoration of normal AV conduction during drug infusion	4 (25)	39 (63)	0.007
Increasing in heart rate (bpm)	18 (11–26)	33 (27–36)	0.01

Values are expressed as the number/total (percentage) of subjects or median (25th-75th percentiles). AV indicates atrioventricular.

## Data Availability

Data supporting the findings of this study are available from the corresponding author upon reasonable request.

## Abbreviations

AV	atrioventricular
ACLS	advanced cardiac life support
TCP	transcutaneous pacing
ED	emergency department
VA	ventricular arrhythmias
